# Evolution of fixed-time AI in dairy cattle in Brazil

**DOI:** 10.21451/1984-3143-AR2018-0020

**Published:** 2018-08-03

**Authors:** Jose Luiz Moraes Vasconcelos, Marcos Henrique Colombo Pereira, Milo Charles Wiltbank, Thiago Guzela Guida, Francisco Rebolo Lopes, Carlos Patricio Sanches, Lucas Furtado dos Santos Pereira Barbosa, Wedson Maria Costa, Anderson Kloster Munhoz

**Affiliations:** 1 Department of Animal Production, FMVZ-UNESP, Botucatu, SP, 18610-000, Brazil; 2 Department of Dairy Science, University of Wisconsin-Madison, Madison, WI, 53706, USA

**Keywords:** FTAI, pregnancy rates.

## Abstract

Various programs have been used to synchronize ovulation of a fertile oocyte, accompanied by fixed-time artificial insemination (FTAI). These programs involve a series of hormonal treatments to achieve four physiologic outcomes: 1) synchronize an ovarian follicular wave; 2) optimize conditions for ovulatory follicle development; 3) synchronize corpus luteum (CL) regression; and 4) synchronize ovulation. This manuscript summarizes studies conducted in Brazil with lactating dairy cows that aimed to increase pregnancy rates to E2/P4-based programs.

## Introduction

Recent reviews have considered the history, physiological basis, and practical use of FTAI for reproductive management of dairy cattle (Baruselli *et al*., 2012; Binelli *et al*., 2014; Wiltbank and Pursley, 2014). This review will examine some specific published research manuscripts that were done with ample statistical power (>200 cows/treatment) and, in our opinion, have been critical for the development of the programs that are in current use for FTAI in dairy cattle in Brazil.

Successful synchronization of ovulation and timed AI involves 4 essential physiological processes: 1) synchronize emergence of a new ovarian follicular wave; 2) optimize the environment for follicular wave development and selection of a single dominant follicle; 3) induce complete CL regression, resulting in low circulating progesterone (P4) near the time of AI; and 4) induce synchronized ovulation, combined with an optimal schedule for FTAI. These programs use various hormones, including: gonadotropin releasing hormone (GnRH), estradiol (E2) derivatives including estradiol benzoate (EB) and estradiol cypionate (ECP), intravaginal (P4) implants, and prostaglandin F_2α_ analogues (PGF). Achieving these physiologic goals using various combinations of hormones has generally resulted in two types of programs, one based primarily on GnRH (Pursley *et al*., 1995, 1997), used in the USA and several other countries, and E2/P4-based programs (Vasconcelos *et al*., 2011b; Wiltbank *et al*., 2011a), used in Brazil and elsewhere. Recent research has altered timing, dosages, and sequences of hormonal treatments, including merging these two types of programs striving to optimize the four principal processes of a synchronization program and thus improve fertility. This manuscript will focus on research done in Brazil to optimize FTAI programs in lactating dairy cattle. Information will be presented in the context of the four physiological processes essential for a successful FTAI program.

## Physiology I: Synchronization of emergence of a new ovarian follicular wave

Understanding follicular waves was essential for initial development of FTAI protocols and their subsequent improvement (Thatcher and Santos, 2007; Wiltbank *et al*., 2011a; Wiltbank and Pursley, 2014). Synchronization of a new follicular wave near the beginning of a FTAI protocol has generally been done by two methods: 1) ovulation of the dominant follicle, usually with GnRH; or 2) inhibition of gonadotropin secretion, usually with EB, leading to regression of the current follicular wave and emergence of a new follicular wave. Neither of these treatments are completely efficient in lactating dairy cows, with only 50-65% of cows ovulating in response to GnRH treatment given at a random stage of the estrous cycle (Vasconcelos *et al*., 1999; Thatcher *et al*., 2002; Giordano *et al*., 2013; Wiltbank and Pursley, 2014) and 25 to 30% of dairy cows ovulating a persistent follicle, due to lack of regression of the previous follicular wave following EB treatment (Monteiro *et al*., 2015; Melo *et al*., 2016). Since it is likely that certain physiological processes, such as synchronized emergence of a new follicular wave, may be more critical in FTAI than during ET programs and therefore results of AI and ET have been compared in some of these studies.

An experiment (Vasconcelos *et al*., 2011b) was done to compare effects of two protocols for synchronization of ovulation (GnRH- *vs*. E2-based protocols) on P4 concentrations and fertility in lactating dairy cows subjected to either FTAI or fixed-time embryo transfer (FTET). A total of 883 lactating Holstein cows (166.2 ± 3.3 days postpartum, yielding 36.8 ± 0.34 kg of milk/day) from eight commercial dairy farms were used. Within each farm, cows were randomly assigned to receive one of the two following treatments for synchronization of ovulation: 1) GnRH Group: day-10 P4 insert (1.9 g of P4; CIDR®) + GnRH, day-3 P4 withdrawal + PGF, day-2: ECP, day 0 FTAI or day 7 FTET (nFTAI = 180; nFTET = 260); and 2) EB Group: Same as above, except EB on day-10 in lieu of GnRH (nFTAI = 174; nFTET = 269). Circulating P4 on day-3 was greater in GnRH than EB treatment (2.89 ± 0.15 *vs*. 2.29 ± 0.15 ng/ml; P < 0.01), but there were no effects of treatments on P4 on day 7 (3.15 ± 0.13 *vs*. 3.03 ± 0.14 ng/ml; P > 0.10). Pregnancy rate at 60 days was higher for FTET compared to FTAI (37.6% [199/529] *vs*. 26.5% [94/354]; P < 0.001). However, there were no effects of GnRH *vs*. EB on synchronized ovulation (87.0% [383/440] *vs*. 85.3% [378/443]), P/AI at 60 days (27.2% [49/80] *vs*. 25.9% [45/174]) or P/ET (38.8% [101/260] *vs*. 36.4% [98/269]), or when only synchronized cows are considered for P/AI at 60 days (35.3% [55/156] *vs*. 33.8% [50/148]) or P/ET (50.7% [115/227] *vs*. 51.3% [118/230]). Thus, either GnRH or EB could be used at the start of the protocol; although GnRH-treated cows had higher P4 during the protocol than those given EB, reproductive performance was similar.

Another study (Pereira *et al*., 2015) evaluated fertility in a FTAI protocol that compared EB versus EB + GnRH at the start of the protocol. For this study a GnRH treatment at the beginning of the protocol was added to a standard Brazilian protocol that was initiated with EB in order to evaluate whether ovulation of a dominant follicle in some cows could improve fertility due to greater synchronization of the follicular wave and greater circulating P4 during growth of the ovulatory follicle. Due to ovulation of a new follicle and consequently a CL, this study also evaluated using two PGF treatments at the end of the protocol to optimize CL regression (discussed in next section). Lactating Holstein cows (n = 1808) were randomly assigned during cool or hot seasons to receive FTAI (day 0) after one of three treatments: Control: CIDR + 2 mg of EB on day-11, PGF on day-4, CIDR withdrawal + 1.0 mg of ECP on day-2, and FTAI on day 0; 2PGF: Identical to the Control protocol, with addition of a second PGF treatment on day-2; and GnRH: Identical to the 2PGF protocol, with addition of 100 µg GnRH on day-11. Pregnancy diagnoses were done 32 and 60 days after FTAI.

Season had major effects on many reproductive measures, with more cool *vs*. hot season cows having a CL at PGF (62.9 *vs*. 56.2%), expressing estrus (86.7 *vs*. 79.9%), ovulating following the protocol (89.7 *vs*. 84.3%), becoming pregnant following the protocol (45.4 *vs*. 21.4%), and having larger ovulatory follicle diameter at AI (15.7 *vs*. 14.8 mm). The GnRH protocol increased percentage of cows with a CL (Control = 56.9%; 2PGF = 55.8%; GnRH = 70.5%) and circulating P4 concentrations on day-4 (Control = 3.28 ± 0.22; 2PGF = 3.35 ± 0.22; GnRH = 3.70 ± 0.21 ng/ml). GnRH also increased P/AI at 32 days (37.3% [219/595]) and 60 days (31% [179/595]) after TAI, compared to Control (30.0% [177/604] and 25.1% [145/604]) with intermediate results for the 2PGF protocol (33.2% [196/609] and 28.0% [164/609]). Positive effects of GnRH treatment on P/AI were only detected during the cool season (Control = 41.0%; 2PGF = 44.2%; GnRH = 50.9%) but not during the hot season (Table 1). In addition, there was only a significant effect of GnRH in cows with low P4 (<3 ng/ml) at the start of the protocol, with no significant effect in cows that had high P4 at the outset. Further, there was an interaction for presence of CL at PGF with follicle diameter; cows with a CL at PGF had greater P/AI if they ovulated larger *vs*. smaller follicles near TAI.

In conclusion, combining GnRH with EB increased fertility, as compared to EB alone, when used at the start of an E2/P4-based protocol, particularly during the cool season and in cows with low P4 at the outset.

**Table 1:** Effects of various treatment protocols on pregnancies per AI (P/AI) during hot *vs*. cool seasons.

		Protocol		
Item^*^	Control	2PGF	GnRH	P Value
P/AI 32d for all cows^1^				
Cool	41.0 (116/283)^b^	44.2 (125/283)^y^	50.9 (148/291)^ax^	0.05
Hot	19.0 (61/321)	21.8 (71/326)	23.4 (71/304)	0.40
P Value	<0.01	<0.01	<0.01	
Combined	30.0 (177/604)^b^	33.2 (196/609)^b,y^	37.3 (219/595)^a,x^	0.02
P/AI D60 for all cows^1^				
Cool	32.9 (93/283)^b^	36.4 (103/283)^ab^	41.6 (121/291)ª	0.09
Hot	16.2 (52/321)	18.7 (61/326)	19.1 (58/304)	0.59
P Value	<0.01	<0.01	<0.01	
Combined	25.1 (145/604)^b^	28.0 (164/609)^a,b^	31.0 (179/595)^a^	0.06
P/AI D32 for synchronized cows^2^				
Cool	46.6 (110/236)^b^	49.0 (120/245)^ab^	55.7 (142/255)^a^	0.11
Hot	22.5 (52/231)	26.0 (60/231)	27.4 (61/223)	0.47
P Value	<0.01	<0.01	<0.01	
Combined	34.7 (162/467)^b^	37.8 (180/476)^y^	42.5 (203/478)ª^x^	0.05
P/AI D60 for synchronized cows^2^				
Cool	37.7 (89/236)^y^	40.4 (99/245)^xy^	45.5 (116/255)^x^	0.20
Hot	19.5 (45/231)	21.7 (50/231)	21.5 (48/223)	0.81
P Value	<0.01	<0.01	<0.01	
Combined	28.7 (134/467)^y^	31.3 (149/476)^xy^	34.3 (164/478)^x^	0.18

* Least square means (n./n.);

1 All inseminated cows;

2 Includes only cows that ovulated to ECP (visible CL at day 7); a,b Within a row = P ≤ 0.05; x, y Within a row = P > 0.05 and P ≤ 0.1. From Pereira *et al*. (2015).

## Physiology II. Optimization of environment for follicular wave development and dominant follicle selection

In dairy cows, a variety of methods have been evaluated to increase fertility in synchronization of ovulation programs, including: increasing P4 concentration during ovulatory follicle development (Bisinotto *et al*., 2010; Martins *et al*., 2011; Wiltbank *et al*., 2011b), increasing length of proestrus (Peters and Pursley, 2003; Pereira *et al*., 2013b), reducing follicle age (Cerri *et al*., 2009; Santos *et al*., 2010), and supplementing estrogen (E2) during proestrus (Cerri *et al*., 2004; Brusveen *et al*., 2009; Souza *et al*., 2011). Enhanced steroid metabolism in lactating dairy cows (Sangsritavong *et al*., 2002; Vasconcelos *et al*., 2003) alters reproductive physiology in dairy cattle (Wiltbank *et al*., 2006), including changes in the preovulatory follicle and ovulated oocyte. For example, there is a decrease in fertility following ovulation of larger follicles, persistent follicles, or follicles that grew when P4 concentrations are low, apparently by reducing embryo quality 1 week after AI (Wiltbank *et al*., 2014). In addition, greater P4 concentrations during the TAI protocol and greater E2 concentrations near time of AI may optimize oviductal and uterine environments, thus improving fertility in high-producing dairy cows (Miller *et al*., 1977; Buhi, 2002).

**Figure 1:**
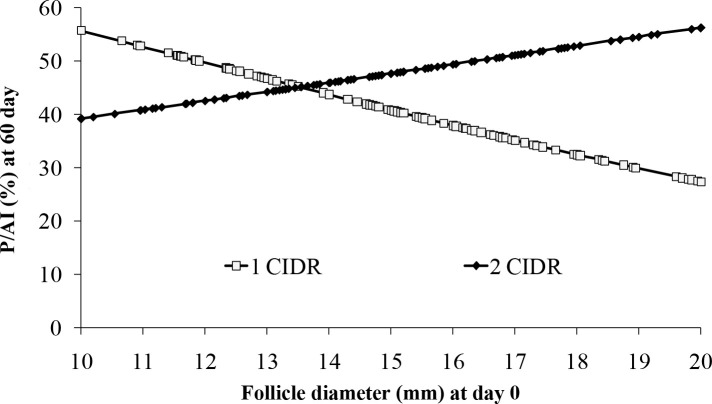
Effects of follicle diameter at time of AI (day 0) on P/AI at day 60 pregnancy diagnosis in dairy cows that ovulated to the protocol (CL on day 7). There was an interaction (P *=* 0.05) between treatment and follicle diameter on P/AI at day 60 apparently due to P/AI decreasing in cows with larger follicles in cows with only 1 CIDR but increasing with follicle size in cows with 2 CIDRs. From Pereira *et al*. (2017a).

Cows without a CL at initiation of FTAI protocols have lower circulating P4 concentrations during development of the preovulatory follicular wave and reduced P/AI after FTAI. An experiment (Pereira *et al*., 2017a) was designed to evaluate effects of increased circulating P4 during preovulatory follicle growth prior to FTAI or FTET, in lactating dairy cows without a CL. Lactating dairy cows with no CL and low circulating P4 (≤1.0 ng/ml) were assigned to a protocol using one or two intravaginal P4 implants (CIDRs) and subjected to FTAI or FTET. The low P4 cows for this experiment were identified on nine farms, of which four utilized FTAI (n = 326 of 1,160 cows examined) and five utilized FTET (n = 445 of 1,396). All cows were synchronized by insertion of one or two P4 implant(s) (CIDR[s]) at start of protocol (day-11) and simultaneous treatment with 2 mg of EB. Seven days later, cows were treated with PGF (day-4) and 2 d later treated with 1.0 mg ECP and CIDR(s) were removed (day-2). Cows received FTAI on day 0 or FTET on day 7. Cows were also randomly assigned to receive either one or two CIDRs from day-11 until day-2 (1CIDR *vs*. 2CIDR). The 2CIDR treatment increased circulating P4 at day-4 (2.18 ± 0.24 *vs*. 1.77 ± 0.23 ng/ml) but had no effect on ovulation at end of protocol (83.6 *vs*. 82.6%) or ovulatory follicle diameter (15.6 ± 0.3 *vs*. 15.3 ± 0.3 mm). If only cows that ovulated to the protocol were included, 1CIDR tended to have lower P/AI than 2CIDR at 32 days (42.8 *vs*. 52.6%; P = 0.10) and 60 days (37.7 *vs*. 48.1%; P = 0.08), with no effect on pregnancy loss. There was an interaction (P = 0.05) between ovulatory follicle diameter and CIDR treatment (Fig. 1) on P/AI (day 60). In cows ovulating larger follicles≥(14 mm) , 2CIDR treatment increased P/AI compared to 1CIDR (53.3 *vs*. 34.9%; P = 0.02) but not in cows ovulating small follicles (<14 mm). There was no effect of treatment on P/ET at 32 days (30.0 *vs*. 32.0%) or 60 days (24.7 *vs*. 25.6%). Thus, these results add evidence to the concept that increased circulating P4 concentrations during preovulatory follicle development may improve P/AI, most likely due to improved oocyte quality in cows that ovulate larger follicles, since there was improved fertility only in cows ovulating larger follicles, with no significant effect of preovulatory P4 concentrations in cows that ovulated smaller follicles or that received FTET.

**Figure 2:**
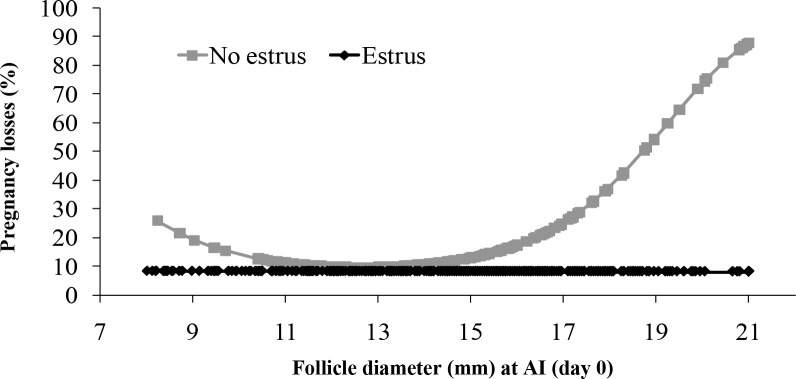
Effects of expression of estrus and follicle diameter at AI (day 0) on pregnancy losses between 32 and 60 days in synchronized dairy cows. No estrus P *=* 0.01, estrus P *=* 0.97. From Pereira *et al*. (2014).

Length of an E2/P4 FTAI protocol has also been evaluated (Pereira *et al*., 2014). Lactating Holstein cows (n = 759) were given a CIDR for either 8 or 9 d with 2 mg EB treatment at beginning, PGF 2 days before CIDR removal, and CIDR removal with 1 mg ECP at 2 days before FTAI. Cows were considered to have their estrous cycle synchronized in response to the protocol by the absence of a CL at AI (D0) and presence of a CL on day 7. Pregnancy diagnoses were performed at 32 and 60 days. Ovulatory follicle diameter at FTAI did not differ, although the 9 d program tended (P = 0.06) to have greater P4 on day 7 in synchronized cows (3.14 ± 0.18 ng/ml) than the 8 days program (3.05 ± 0.18 ng/ml). Although P/AI at 32 days (8 days = 45% [175/385] *vs*. 9 days = 43.9% [166/374]; P = 0.77) and at 60 days (8 days = 38.1% [150/385] *vs*. 9 days = 40.4% [154/374]; P = 0.52) was not different, the 9 days program had lower (P = 0.04) pregnancy losses (7.6% [12/166]) than the 8 days program (14.7% [25/175]). Cows in the 9 days program were more likely (P < 0.01) to be in estrus (72.0% [269/374]) than those in the 8 days program (62% [240/385]). Expression of estrus improved estrous cycle synchronization (97.4% [489/501] *vs*. 81% [202/248]; P < 0.01), P4 concentrations at day 7 (3.22 ± 0.16 *vs*. 2.77 ± 0.17 ng/ml; P < 0.01), P/AI at 32 days (51.2% [252/489] *vs*. 39.4% [81/202]; P < 0.01) and at 60 days (46.3% [230/489] *vs*. 31.1% [66/202]; P < 0.01), and it decreased pregnancy loss (9.3% [22/252] *vs*. 19.8% [15/81]; P < 0.01), compared to cows not detected in estrus. Those not detected in estrus with small (<11 mm) or large follicles (>17 mm) had greater pregnancy loss (P = 0.01); however, in cows detected in estrus, there was no effect (P = 0.97) of follicle diameter on pregnancy loss (Fig. 2). In conclusion, increasing the length of the protocol for FTAI increased the percentage of cows detected in estrus and reduced pregnancy losses.

A final experiment was done to compare two protocols that increase P4 during preovulatory follicle development (Pereira *et al*., 2017b). One treatment utilized two intravaginal P4 implants (CIDR), whereas the other utilized GnRH at start of the protocol. Lactating Holstein cows that had been diagnosed as non-pregnant were randomly assigned to receive FTAI following one of two treatments (n = 1,638 breedings): GnRH - CIDR + 2 mg EB + 100 µg GnRH on day-11, PGF on day-4, CIDR withdrawal + ECP + PGF on day- 2, and TAI on day 0; or 2CIDR - Two CIDR + EB D- 11, one CIDR withdrawn + PGF on day-4, second CIDR withdrawn + ECP + PGF on day-2, and FTAI on day 0. There was no effect of treatments (P > 0.10) on P/AI or pregnancy loss. Various physiological measurements associated with greater fertility were reduced (P < 0.01) in cows with an elevated body temperature (compared to those without), including: cows with CL at PGF (decreased 7.9%), ovulatory follicle diameter (decreased 0.51 mm), expression of estrus (decreased 5.1%), and ovulation near FTAI (decreased 2.8%). There were a greater proportion of cows (30.2%; P < 0.01) with a CL at PGF in the GnRH (74.1% [570/763]) than 2CIDR treatment (56.9% [434/763]). However, circulating P4 was greater (P = 0.05) at PGF treatment (day-4) for cows treated with 2CIDR (4.26 ± 0.13 ng/ml) than GnRH (3.99 ± 0.14 ng/ml). Thus, these two protocols yield similar fertility (Table 2), although that was likely due to somewhat different physiological alterations. Exogenous GnRH increased the proportion of cows with a CL at PGF; however, the 2CIDR protocol increased circulating P4 under all circumstances.

In conclusion, optimizing the length of the protocol and increasing circulating P4 during the protocol are strategies currently used to increase fertility during FTAI protocols in dairy cattle in Brazil. Similarly, with high-yielding dairy cattle in other countries, increasing P4 during the FTAI protocol can increase fertility (Wiltbank *et al*., 2014).

**Table 2:** Effects of two different protocols and rectal temperature on ovulation to ECP, P/AI at the 32 or 60 days pregnancy diagnosis, and pregnancy loss from 32 to 60 days. The two protocols were both designed to increase circulating P4 during the protocol using either GnRH at beginning (GnRH) or 2 CIDRs rather than only 1 CIDR during the protocol (2CIDR).

Rectal temperature (ºC)				P value	
Item^*^	<39.1	≥39.1	Temp.	Prot.	Interaction
Synchronized cows^1^					
GnRH	88.1 (355/402)	83.3 (304/361)	0.09	0.93	0.34
2CIDR	86.2 (359/415)	84.8 (298/348)			
P/AI 32 days for all cows^2^					
GnRH	39.7 (162/402)	26.2 (98/361)	<0.01	0.64	0.42
2CIDR	38.9 (164/415)	29.3 (105/348)			
P/AI 60 days for all cows^2^					
GnRH	34.0 (138/402)	22.0 (81/361)	<0.01	0.91	0.79
2CIDR	33.6 (141/415)	22.9 (81/348)			
P/AI 60 days for synchronized cows^3^					
GnRH	38.9 (138/355)	26.6 (81/304)	<0.01	0.86	0.98
2CIDR	39.3 (141/359)	27.2 (81/298)			
Pregnancy loss (32-60 days)^3^					
GnRH	14.8 (24/162)	17.4 (17/98)	0.09	0.48	0.34
2CIDR	14.0 (23/164)	22.9 (24/105)			

* Least square means (n./n.);

1 Based on presence of CL on day 7, as determined by ultrasound;

2 Includes all inseminated cows;

3 Includes only cows that ovulated to ECP (visible CL on day 7 after FTAI). From Pereira *et al*. (2017b).

## Physiology III. Inducing complete regression of CL and low P4 near TAI

Low P4 concentrations near the time of AI is essential for optimal fertility in both GnRH-based (Souza *et al*., 2005; Brusveen *et al*., 2009; Martins *et al*., 2011; Wiltbank *et al*., 2015) and E2/P4-based (Pereira *et al*., 2013b; Monteiro *et al*., 2015) protocols. Various methods have been used to ensure lower P4 near FTAI, including performing treatments with PGF prior to removal of the intravaginal P4 implant in E2/P4-based TAI programs (Meneghetti *et al*., 2009; Peres *et al*., 2009; Pereira *et al*., 2013b) and treatment with a second PGF, generally 24 h after the first PGF treatment (Brusveen *et al*., 2009; Pereira *et al*., 2015; Wiltbank *et al*., 2015; Melo *et al*., 2016). Earlier treatment with PGF should allow more time for CL regression and subsequent reductions in circulating P4 that could be critical for fertility in cows with a CL during an E2/P4-based program.

An experiment (Pereira *et al*., 2013b) investigated P4 concentrations and fertility comparing treatment with PGF at two times in an E2/P4-based FTAI and FTET program in lactating dairy cows. A total of 1,058 lactating Holstein cows, primiparous (n = 371) and multiparous (n = 687), yielding 34.1 ± 10.4 kg of milk/d were randomly assigned to receive treatment with PGF on either day-3 or day-2 of the following protocol: day-10: 2 mg EB+CIDR; day-2 CIDR removal + 1.0 mg ECP; day 0 - FTAI or day7 - FTET. Only cows with a CL on day 7 received an embryo and all cows received GnRH at time of FTET. Pregnancy diagnoses were performed at 28 and 60 days. Fertility (P/AI or P/ET) was affected by breeding technique (AI *vs*. ET) and time of PGF treatment (day-3 *vs*. day-2), for FTAI (32.9% [238] *vs*. 20.6% [168]) and FTET (47%[243] *vs*. 40.7% [244]) at 28 days, and 60 days for FTAI (30% [238] *vs*. 19.2% [168]) and FTET cows (37.9% [243] *vs*. 33.5% [244]). Circulating P4 on day 0 altered fertility in FTAI with greater P/AI in cows with P4 < 0.1 ng/ml compared to cows with P4 ≥ 0.1 ng/ml, and in FTET with greater P/ET in cows with P4 < 0.22 ng/ml compared to cows with P4 ≥ 0.22 ng/ml (Table 3). Treatment with PGF at day-3 increased percentage of cows with P4 < 0.1 ng/ml on day 0 (39.4 *vs*. 23.2%). Reducing the interval between PGF and FTAI from 72 to 48 h in dairy cows dramatically reduced fertility in cows bred by FTAI and had a subtle negative effect in cows that received FTET. Earlier PGF treatment benefits were most likely mediated through improvements in gamete transport, fertilization, or early embryo development, with other effects of earlier PGF manifest after ET on day 7.

It is critical that FTAI programs have low P4 concentrations near FTAI. This can be done in various ways: increasing the dose of PGF, when cloprostenol was used (Giordano *et al*., 2013), increasing number of PGF treatments (Pereira *et al*., 2015; Wiltbank *et al*., 2015), particularly in programs with GnRH at beginning of protocol, and an increased interval from PGF to FTAI (Pereira *et al*., 2013b) to allow sufficient time for circulating P4 to reach low concentrations.

**Table 3:** Effects of P4 concentrations on day 0 (at AI or 7 days before ET) at day 60 pregnancy diagnosis in lactating dairy cows after FTAI or FTET.

Progesterone (ng/ml) on day 10				
Item^1^	≤0.09	0.10-0.21	≥0.22	P-value
TAI P/AI at Day 60				
PGF day-3	39.4 (36/85)	27.5 (8/26)	24.0 (12/45)	_
PGF day-2	23.2 (15/54)	15.1 (8/45)	14.6 (4/22)	_
Combined^2^	34.1 (51/139)^ax^	20.2 (16/71)^b^	21.4 (16/67)^y^	0.05
TET P/ET at day 60				
PGF day-3	46.8 (37/77)	44.2 (23/52)	25.3 (12/49)	_
PGF day-2	40.0 (24/58)	46.0 (33/73)	20.5 (9/50)	_
Combined^2^	43.8 (61/135)^a^	45.3 (55/125)^a^	22.9 (21/99)^b^	0.0006

1 Each value includes least-squares means % (no./no.);

2 Combined values of treatments to determine the effect of P4 at day 0 on P/AI or P/ET; a, b Within a row = P < 0.05; x, y Within row = P > 0.05 and P ≤ 0.01. From Pereira *et al*. (2013b).

## Physiology IV: Synchronizing time of ovulation and optimizing fertility to TAI

Serum E2 concentrations at FTAI were positively correlated with ovulatory follicle diameter (Vasconcelos *et al*., 2001; Perry *et al*., 2005). Furthermore, cows ovulating smaller follicles following GnRH treatment were more likely to have reproductive failure (Perry *et al*., 2005; Pereira *et al*., 2013a; Vasconcelos *et al*., 2013). There is likely an interaction among preovulatory follicle diameter, and optimal hormonal environment (manifested by expression of estrus) and establishment and maintenance of pregnancy (Jinks *et al*., 2013; Perry *et al*., 2014).

Studies comparing induction of ovulation using GnRH *vs.* ECP (Souza *et al*., 2009) or ECP *vs*. EB (Melo *et al*., 2016) were done in Brazil. All of these ovulation-inducing hormones yielded similar fertility. Therefore, despite differences in interval from treatment to ovulation, they were similarly effective in synchronizing ovulation and produced similar fertility.

**Figure 3:**
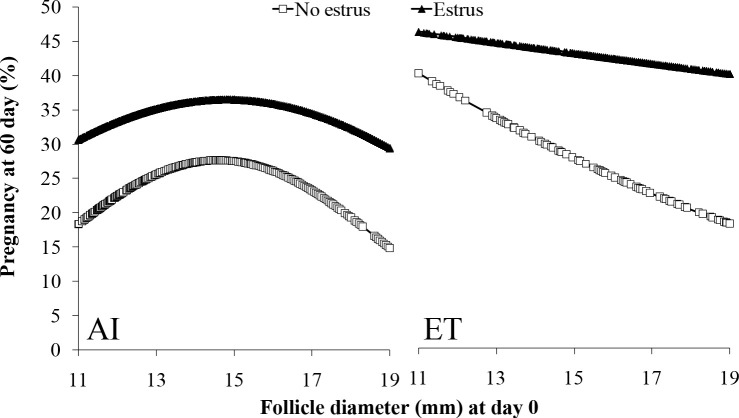
Effects of follicle diameter on day 0 on P/AI (AI) or P/ET (ET) at day 60 in cows that did or did not display estrus. P/AI: No Estrus P < 0.01; Estrus P < 0.01. P/ET: No Estrus P = 0.08; Estrus P = 0.40. From Pereira *et al*. (2016).

**Figure 4:**
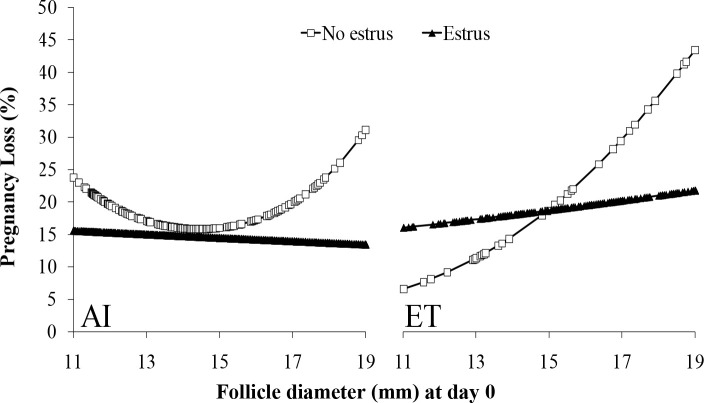
Effects of follicle diameter on day 0 on pregnancy losses (after AI or ET) between day 32 and day 60 in cows that did or did not display estrus or did not display estrus AI: No Estrus, P. 0.13; Estrus, P = 0.46. ET: No Estrus, P = 0.05; Estrus P = 0.45. From Pereira *et al*. (2016).

**Figure 5:**
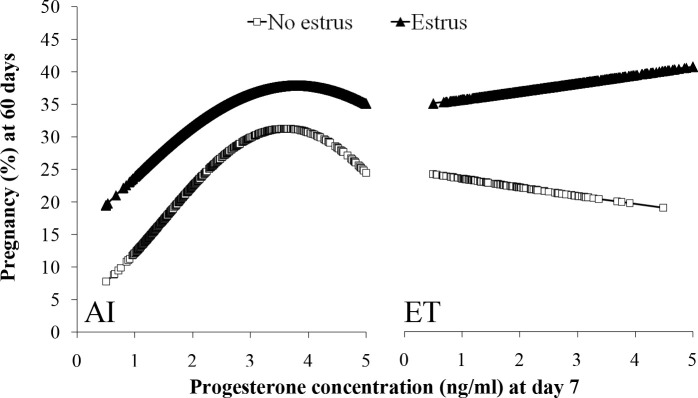
Effects of P4 concentration on day 7 on P/AI or P/ET at day 60 in cows that did or did not display estrus. AI: No Estrus, P = 0.02; Estrus, P = 0.01. ET: No Estrus, P = 0.76; Estrus P = 0.52. From Pereira *et al*. (2016).

In lactating dairy cows synchronized with E2/P4 protocols that used ECP for induction of ovulation, estrus expression dramatically affected fertility. A large study (Pereira *et al*., 2016) evaluated expression of estrus and fertility in FTAI (n = 5,430) or FTET (n = 2,003) programs. Ovarian ultrasonography (US) was performed on day0 (time of AI) and day 7 to determine ovulatory follicle diameter and ovulation. Only cows with a visible CL on day 7 were used. At CIDR removal, all cows received a tail- head device for detection of estrus and were considered in estrus when the paint of the device was completely removed by day 0. Circulating P4 concentrations were evaluated on day 7. At pregnancy diagnosis on day 32, cows with expression of estrus had increased (P < 0.01) P/AI (no estrus = 25.5% [222/846] *vs*. estrus = 38.9% [1785/4584]) and P/ET (no estrus = 32.7% [193/606] vs. estrus = 46.2% [645/1397]). Similarly, at pregnancy diagnosis on day 60, expression of estrus increased (P < 0.01) P/AI (no estrus = 20.1% [179/846] *vs*. estrus = 33.3% [1530/4584]) and P/ET (no estrus = 25.1% [150/606] *vs*. estrus = 37.5% [525/1397]). Pregnancy loss was lower (P = 0.01) in cows that expressed estrus in FTAI (no estrus = 20.1% [43/222] *vs*. estrus = 14.4% [255/1785]) and FTET (no estrus = 22.7% [43/193] *vs*. estrus = 18.6% [120/645]) compared to cows with no estrus. Independent of expression of estrus, P/AI was reduced in cows ovulating either too small or too large of follicles (Quadratic effect; P < 0.01; Fig. 3). There was no effect (P = 0.40) of ovulatory follicle diameter on P/ET in cows that expressed estrus; however, cows that did not express estrus tended to have lower (P = 0.08) P/ET if they ovulated larger follicles. In cows detected in estrus, follicle diameter did not affect pregnancy loss (AI: P = 0.46; ET: P = 0.45), but cows not detected in estrus and ovulating larger follicles tended to have greater pregnancy loss after FTAI (P = 0.13) and had greater pregnancy loss after FTET (P = 0.05; Fig. 4). There was a positive effect of day 7 circulating P4 concentrations on P/AI (P < 0.02), independent of estrus (Fig. 5). In contrast, there was no effect (P > 0.5) of circulating P4 concentration on day 7 on P/ET. Thus, expression of estrus during protocols for FTAI or FTET was associated with an increase in fertility and reduction in pregnancy loss. During FTAI programs, optimizing follicle diameter and increasing circulating P4 on day 7 after AI were also associated with increased fertility, independent of expression of estrus. However, in cows with FTET, the association of fertility with either ovulatory follicle diameter or P4 on day 7 was less dramatic and seemed to be related to whether cows expressed estrus.

## Direct comparison of GnRH *vs*. E2/P4-based TAI programs

**Figure 6:**
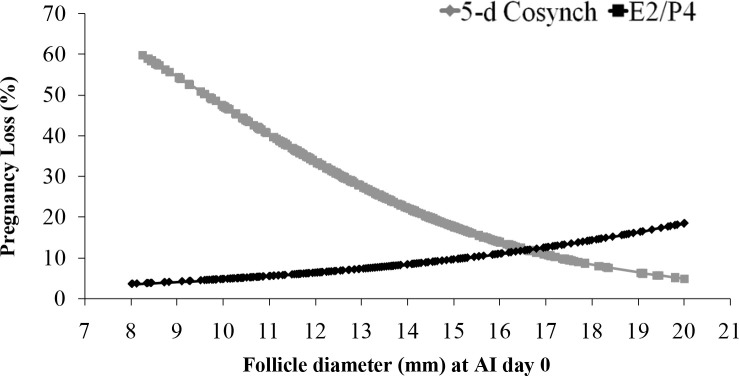
Effect of follicle diameter at AI on pregnancy loss between day 32 and day 60 in cows subjected to either E2/P4 or 5-days Cosynch protocols and had their cycle synchronized (P4 ≤ 0.4 ng/ml at day 0 and P ≥ 1.0 ng/ml at day 7) 5-days Cosynch (P *=* 0.04): y = -0.276x +2.4648; E2/P4 (*P* = 0.32): y = 0.01063x - 3.8241. From Pereira *et al*. (2013a).

As discussed above, FTAI programs based on GnRH have a somewhat different physiology than E2/P4-based FTAI programs, despite similar physiologic and practical goals. One experiment (Pereira *et al*., 2013a) was designed to directly compare a GnRH-based to an E2/P4-based protocol for estrous cycle synchronization and FTAI. For this experiment, a 5 days GnRH protocol was compared to the standard E2/P4-based program, since both are designed for synchronization of ovulation and a reduction in the interval from follicular emergence to ovulation in cows with a synchronized follicular wave. A total of 1,190 lactating Holstein cows, primiparous (n = 685) and multiparous (n = 505), yielding 26.5 ± 0.30 kg of milk/day at 177 ± 5.02 DIM were randomly assigned to one of the following programs: 5-days Cosynch protocol (day-8: CIDR + GnRH, day-3: CIDR removal + PGF, day-2: PGF, day 0: FTAI+GnRH); or E2/P4 protocol (day-10: CIDR + EB, day-3: PGF, day-2: CIDR removal + ECP, day 0: FTAI). Rectal temperature and circulating P4 concentration were measured on the day-3, -2, 0 (FTAI) and day 7. The estrous cycle was considered synchronized when P4 wa≥s 1.0 ng/m l on day 7 in cow that had previously undergone luteolysis (P4 ≤ 0.4 ng/ml on day 0). To evaluate effects of heat stress, cows were classified by number of heat stress events, either 0, 1, or 2+ measurements of elevated body temperature (≥39.1ºC). Pregnancy success (P/AI) was determined at 32 and 60 days after FTAI. Cows in the 5- days Cosynch protocol had increased (P < 0.01) circulating P4 concentrations at PGF treatment (2.66 ± 0.13 *vs*. 1.66 ± 0.13 ng/ml). Cows in the E2/P4 protocol were more likely (P < 0.01) to be detected in estrus (62.8 *vs*. 43.4%) compared to cows in a 5-days Cosynch, and expression of estrus improved (P < 0.01) P/AI in both treatments. Cows in the 5-days Cosynch protocol had greater (P = 0.02) percentage of synchronized cycles (78.2%), compared to cows in the E2/P4 protocol (70.7%). On day 60, the E2/P4 protocol tended (P = 0.07) to improve P/AI (20.7 *vs*. 16.7%) and reduced (P = 0.05) pregnancy loss from 32 to 60 days (11.0 *vs*. 19.6%), compared to 5-days Cosynch protocol. In cows with a synchronized cycle, the E2/P4 protocol had greater (P = 0.03) P/AI (25.6 *vs*. 17.7%) on day 60 and lower pregnancy loss (P = 0.01) from day 32 to day 60 (6.7 *vs*. 21.7%) compared to cows in the 5-days Cosynch protocol. Follicle diameter affected (P = 0.04) pregnancy loss from 32 to 60 days only in cows in the 5-days Cosynch protocol, with smaller follicles resulting in greater pregnancy loss (Fig. 6). P/AI at day 60 was different (P = 0.01) between protocols in the cows with two or more measurements of heat stress (5-days Cosynch = 12.2% *vs*. E2/P4 = 22.8%), but not in cows without or with only one heat stress measurement (P = 0.6). In conclusion, the 5-days Cosynch protocol apparently produced better estrous cycle synchronization than the E2/P4 protocol but did not improve P/AI. The potential explanation for these results is that increased E2 concentrations during the periovulatory period can improve pregnancy success and pregnancy maintenance; furthermore, this effect appeared to be greatest in heat-stressed cows, apparently with lower circulating E2 concentrations.

## Discussion

Research on FTAI in lactating dairy cows in Brazil during the last 20 years has highlighted some key physiological principles that are important for improving fertility to FTAI and FTET programs. First, slight elevations in P4 near AI can reduce fertility. This was illustrated by the finding that an earlier treatment with PGF (i.e. 1 day prior to removal of intravaginal P4 implant; day-3 *vs*. day-2) allowed greater time for regression of the CL, lower P4 at CIDR removal and at FTAI, and greater fertility. Greater P4 concentrations at FTAI (>0.1 ng/ml) were clearly associated with reduced fertility in FTAI cows. In ET cows, fertility improvement after earlier regression of the CL was less dramatic than after AI, with no reduction in fertility to FTET until circulating P4 concentrations near ovulation reached >0.21 ng/ml. Thus, it appears that there is a dramatic effect on fertility of earlier PGF treatment that was likely mediated through more complete CL regression which likely improved gamete transport, fertilization, or early embryo development with more subtle effects of earlier PGF treatment in FTET programs likely mediated through changes in the uterine or hormonal environment after day 7 (time of ET).

Second, the absence of a CL and low circulating P4 at initiation of a protocol also appears to be a problem for dairy herds in Brazil. Two methods for increasing P4 during E2/P4-based FTAI protocols in lactating dairy cows that were discussed in this manuscript are: 1) combine GnRH with the EB at the beginning of the protocol (Pereira *et al*. 2015) or 2) utilize two intravaginal P4 implants rather than only one implant during the protocol (Pereira *et al*. 2017a). Both of these strategies have resulted in greater fertility in lactating cows that do not have a CL at the start of the protocol.

Third, follicle diameter was linked to P/AI in several studies. Ovulation of an undersized follicle was generally associated with reduced P/AI, reduced E2 concentrations, an increased incidence of short luteal phases (Vasconcelos *et al*., 2001), and sometimes increased pregnancy loss (Perry *et al*., 2005). In contrast, ovulation of an oversized follicle can also be associated with reduced P/AI, perhaps due to ovulation of a persistent dominant follicle (Townson *et al*., 2002, Bleach *et al*., 2004, Cerri *et al*., 2009). In a number of these studies, follicle size in cows that did not display estrus was related to P/AI and P/ET at either the 32 or 60 days pregnancy diagnoses.

Fourth, the critical effect of estrus as a predictor of fertility in E2/P4-based FTAI programs has been clearly illustrated. Expression of estrus requires low circulating P4 concentrations plus increased circulating E2 of a sufficient magnitude and duration (Allrich, 1994). We measured P4 near the time of AI in a subset of cows (n = 2372; Pereira *et al*., 2016); there was a difference (P < 0.01) in circulating P4 in cows that expressed (0.15 ± 0.03 ng/ml) versus cows that did not express estrus (0.19 ± 0.03 ng/ml). Although relatively small in magnitude, this may account for a lack of behavioral estrus in some cows. In cows that had their cycle synchronized, expression of estrus was associated with an increase in P/AI and P/ET at 32 and 60 days and reduced pregnancy losses between 32 and 60 days of pregnancy. Similar results were reported in previous studies in dairy cows using various FTAI protocols. For example, using Heatsynch protocols, cows that displayed estrus after the ECP had greater P/AI (42.5% [306]) than cows not in estrus (21.1% [71]) at FTAI (Cerri *et al*., 2004). Therefore, expression of estrus was a predictor of fertility and pregnancy loss in both FTAI and FTET programs.

Finally, one of the largest problems for reproductive management programs of lactating dairy cows in Brazil is the reduced P/AI that occurs due to heat stress (Vasconcelos *et al*., 2006, 2011a, b, c). Plasma E2 concentrations are reduced by heat stress in dairy cows (Wolfenson *et al*., 1995, 1997; Wilson *et al*., 1998) and E2 supplementation may improve fertilization, subsequent embryonic development, and pregnancy maintenance. Pregnancy loss was reduced or tended to be reduced in cows with expression of estrus (Pereira *et al*., 2013a, 2016), those with greater circulating E2 concentrations near FTAI (Souza *et al*., 2007, 2011; Hillegass *et al*., 2008), and cows that had increased length of proestrus (Ribeiro *et al*., 2012). In these studies, cows detected in estrus had decreased pregnancy losses, irrespective of preovulatory follicle diameter.

In addition, the P4 concentration at day 7 after AI was associated with P/AI (quadratic effect), independent of estrus expression, but was not related to P/ET (Demetrio *et al*., 2007). Higher P4 concentrations may be indicative of a better CL, due to enhanced follicle and oocyte health or physiological function.

## Conclusions

Based on field experiences and the results reported in these studies, heat stress is one of the major factors that reduce fertility in dairy cows during FTAI protocols in Brazil as demonstated by cows with an increased body temperature≥3(9.1ºC) having large reductions in P/AI. In addition, fertility can be substantially altered by the hormonal concentrations during the protocol, when circulating P4 needs to be elevated, and near the end of the protocol, when P4 needs to be basal and circulating E2 needs to be elevated. Evidence was provided that increasing circulating P4 during preovulatory follicle development improved P/AI, particularly in cows with low P4 at the start of the protocol. This was done by inducing ovulation (with exogenous GnRH) at the beginning of an E2/P4-based protocol, or by inserting a second CIDR at the beginning of the protocol. Near the end of the protocol, treatment with PGF one day before removal of the intravaginal P4 implant, reduced circulating P4 near FTAI and increased fertility following FTAI and FTET. It also appears that increasing E2 concentrations prior to AI can improve pregnancy success and pregnancy maintenance as evidenced by the dramatic effects of expression of estrus on P/AI, P/ET, and pregnancy loss. In addition, increasing the length of the protocol for FTAI increased the percentage of cows detected in estrus and decreased pregnancy loss. Following FTAI programs, increasing circulating P4 7 days after AI was associated with increased fertility, independent of expression of estrus. In cows with FTET, the association of fertility with either preovulatory follicle diameter or circulating P4 concentrations at time of ET (day 7) was less dramatic and seemed to be related to whether cows expressed estrus. Thus, during the last two decades FTAI protocols have been improved and are likely to continue to be improved for dairy cows in Brazil by focusing on increasing synchronization of follicular waves, optimizing hormonal concentrations during specific stages of the protocol, and by improving percentage of cows ovulating in a synchronized time period at the end of the protocol.
